# The Importance of Community Engagement and Research Translation within the NIEHS Superfund Research Program

**DOI:** 10.3390/ijerph16173067

**Published:** 2019-08-23

**Authors:** Brittany A. Trottier, Danielle J. Carlin, Michelle L. Heacock, Heather F. Henry, William A. Suk

**Affiliations:** National Institute of Environmental Health Sciences, 111 TW Alexander Drive, Durham, NC 27709, USA

**Keywords:** community engagement, environmental health education, prevention and intervention activities, research translation

## Abstract

The National Institute of Environmental Health Sciences Superfund Research Program (SRP) funds university-based, solution-oriented research to understand how hazardous substances contribute to disease and how to prevent exposures to these hazardous substances. A unique aspect of the SRP is that, beyond the biomedical, environmental sciences, and engineering research projects, SRP-funded centers are required to include community engagement to build partnerships with affected communities and research translation to communicate and facilitate the use of research findings. The SRP views both as effective ways to inform and advance science for protection of public health. The purpose of community engagement within the centers is to ensure bidirectional communication between the researchers and the community, identify best practices and activities in community engagement for prevention and intervention activities, enhance knowledge, and support the needs of the communities impacted by hazardous waste sites. The SRP views research translation as communicating and facilitating the use of research findings emanating from the center in a manner most appropriate for their application and for the advancement of a center’s research objectives. The SRP has a strong history of seeking opportunities to work with communities and stakeholders, by translating and sharing research findings in an impactful and informative manner with long-lasting benefits to improve public health.

## 1. Introduction

The Superfund Research Program (SRP) was established in 1986 by the Superfund Amendments and Reauthorization Act (SARA) [1]. Housed within the National Institute of Environmental Health Sciences, an Institute of the National Institutes of Health, the SRP supports research to discover approaches to protect the public from exposure to hazardous substances found in water, soil, and air found at hazardous waste sites. The SRP has four mandates that research activities and training are expected to address: (1) Advanced techniques for the detection, assessment, and evaluation of the effect of hazardous substances on human health; (2) Methods to assess the risks to human health presented by hazardous substances; (3) Methods and technologies to detect hazardous substances in the environment; and (4) Basic biological, chemical, and physical methods to reduce the amount and toxicity of hazardous substances [2]. In addition, the SRP strives to push the boundaries by supporting science using the newest technologies and challenging current scientific paradigms.

Since its inception, the SRP has funded university-based centers which focus on basic biological, environmental, and engineering processes to find practical solutions to clean up, prevent exposure to, and determine human health effects of hazardous substances. The overarching goal of these centers is to improve public health by supporting multidisciplinary research that includes at least two biomedical projects and two environmental science and engineering projects. Centers are also required to include cores with specific foci (e.g., data management and analysis), as well as research translation and community engagement components. This summary specifically focuses on how SRP centers proactively communicate their scientific accomplishments and findings to stakeholders through community engagement and research translation.

## 2. Importance of Community Engagement

The SRP asks that their grantees ensure their research findings are clearly communicated to individuals and communities and that these communities have an opportunity to benefit from the research. The SRP accomplishes this by requiring each center to have a Community Engagement Core (CEC). The SRP defines community engagement as the bidirectional interaction and communication between community stakeholders and its centers. The SRP and NIEHS view community engagement as an effective way to inform and advance science for public health protection. Community Engagement Core activities build from the research and expertise of the center’s scientific projects and cores.

The SRP is also aware that communities face many challenges when living near hazardous waste sites, not only by having to understand the health impacts of hazardous substances but also determining ways to prevent or reduce exposures. Through the CECs, the centers work with communities impacted by hazardous substances to identify opportunities for prevention and intervention activities, thus providing potential solutions to the communities to reduce or mitigate the effect of exposure to hazardous substances. In its most recent request for applications and drawing from its fourth mandate [3], the SRP refers to prevention/intervention as “basic biological, chemical, and physical methods to reduce the amount and toxicity of hazardous substances,”. Examples of prevention and intervention activities with communities might include: capacity building; developing tools/resources to enhance environmental health literacy and knowledge of exposures in the target community; developing culturally appropriate prevention and intervention communication strategies tailored for the specific community; and partnering with research projects to share expertise and/or assistance in understanding risks from pathways of concern identified by the community. Several SRP CECs have led successful activities to engage communities in prevention and intervention of harmful substances to improve their health.

### 2.1. Development of Tools/Resources to Enhance Environmental Health Literacy and Knowledge of Exposures

Many SRP centers are involved in developing tools and resources to enhance environmental health literacy and knowledge of exposures in the community. For example, Dartmouth College and Boston University SRP centers have developed websites for communities to access information on the sources of exposure, hazards of exposures, and ways to lower their exposure and provide a place for concerned communities to ask questions about environmental issues in their neighborhoods [4,5]. SRP centers also create educational handouts that clearly communicate the risks of specific environmental exposures and strategies to reduce exposures. The University of Kentucky SRP Center’s CEC created handouts, based on their center’s research on reducing hazardous substance exposure through diet, that they share when providing nutritional education in Kentucky’s Appalachia region [6]. The University of Arizona SRP Center creates infographics for community gardeners that increase environmental health literacy through data sharing and visualization of environmental quality reports via community events and mail [7]. The Duke University SRP Center CEC also provides various sources of information and resources (website, educational materials, newsletters) for community gardeners in North Carolina on the sources and health impacts of soil contaminants, ways to reduce exposure, and how to get their soil tested [8]. The University of Washington SRP Center CEC develops educational videos, trainings, and various educational print materials to warn the public about the dangers of fishing in contaminated areas of the Lower Duwamish Waterway and inform them of the health risks of exposure to the known contaminants in the fish [9]. The CEC at University of Louisville Superfund Center holds community environmental quality knowledge meetings with the local community residents to enhance their environmental health literacy on what is in their environment, what they are exposed to, and how to prevent it.

### 2.2. Development of Culturally Appropriate Prevention and Intervention Communication Strategies

Because communities vary in ethnicity, race, gender, age, or resident status, it is a challenge to develop a single prevention message that works for all. The key to community engagement is developing culturally sensitive and relevant materials and communication strategies to increase the reach of prevention and intervention activities. The SRP CECs overcome this issue by providing tailored and culturally relevant messaging and activities to each stakeholder they work with. Several SRP centers have developed different communication strategies to work with tribal nations and other diverse communities to address their environmental health concerns and connect their cultural beliefs. The Brown University SRP Center launched “The Namaus (All Things Fish) Project” with the Narragansett tribe in Rhode Island to work with the tribe to facilitate informed decision-making regarding fish consumption and fish contamination that was culturally relevant and based on both scientific and local knowledge [10]. The Northeastern University SRP Center uses an innovative mobile application developed by the Silent Spring Institute, called the Digital Exposure Report-Back Interface (DERBI), to provide research results to study participants, in both English and Spanish. The messages include details on the environmental contaminants found in their bodies and how participants can reduce the exposure to these contaminants [11].

### 2.3. Partnership with Research Projects to Share Expertise and Assistance in Understanding Risks of Exposure

The CECs often partner with research projects within their center to lend their expertise with communities and leverage other researcher expertise to share risks of exposure within the community. For example, the Oregon State University SRP Center CEC has paired with multiple research projects within their center to engage tribal communities in evaluating and reducing exposure, using a community-engaged approach [12,13]. The Boston University and University of Iowa SRP Centers’ projects and CECs collaborated with each other to establish partnerships for community-based environmental exposure research and to develop tools and methods to report back to the community members, a strategy that combined multiple areas of expertise to best reach a community affected by polychlorinated biphenyl and heavy metal contamination [14]. These prevention and intervention activities have helped to empower impacted communities to be active participants in decisions to reduce the amount and toxicity of hazardous substances in their homes, schools, communities, and environment. In addition, SRP CECs strive to build long-lasting relationships and trust with the impacted communities and/or community-serving organizations (e.g., local government groups, tribal councils, community service groups focused on educating the community, and nongovernmental organizations working closely with a community). The CECs also acknowledge the importance of engaging the community in sharing research findings and have developed innovative strategies to communicate scientific results in a clear and culturally appropriate manner. In addition, grantees can replicate successful engagement strategies with new communities to expand the reach of the center [15].

## 3. Importance of Research Translation

The Superfund Research Program is also committed to fostering research translation of scientific accomplishments to support its mandates. The SRP views research translation as playing a critical role in encouraging accurate and timely use of research outcomes emanating from the centers. Each SRP center facilitates research translation by maintaining effective communication with the NIEHS SRP, within the center, and establishing partnerships with government agencies, administering technology transfer, and disseminating information to other stakeholders and end-users. These efforts are led by a Center’s Research Translation Coordinator, who is assigned to take the center’s research findings and accomplishments and translate the research to the center’s various stakeholders.

### 3.1. Communicating within SRP

The centers are involved in multiple activities to communicate and translate research findings to NIEHS SRP staff and facilitate cross-center communication including networking with each other, coordinating various activities, and keeping each other aware of what they are doing to promote openness for partnerships. This is facilitated by a monthly webinar that all centers use to interact and share highlights from the month. Centers also facilitate this communication through various means. For instance, the Columbia University SRP Center hosts monthly seminar/webinars that they have open to all other SRP centers and other interested parties where they not only feature Columbia SRP Center research but also invite other experts from SRP centers and areas of research pertaining to the SRP mission [16]. The University of California San Diego SRP Center facilitates this by disseminating their high impact publications via their website, Twitter, and press releases.

### 3.2. Partnerships with Government Agencies

SRP centers establish and develop strong relationships with federal, state, local, and tribal agencies that are involved in protecting human health and the environment. These partnerships guarantee that stakeholders have access to the valuable resources developed by the center and that the center’s investigators have feedback from their government counterparts. This ensures that the findings from the centers are communicated and utilized by the appropriate government agencies. Investigators at the University of California- Berkeley SRP Center developed a relationship with the Environmental Protection Agency and the International Agency for Research on Cancer, and contribute to reports that pertain to their expertise, including the NIEHS/EPA Children’s Environmental Health and Disease Impact Report and the IARC Monograph for the carcinogenicity of benzene. The University of New Mexico SRP Center has formed partnerships with tribal agencies, including the Navajo Nation EPA. They also maintain partnerships with the U.S. EPA and are often invited to give research updates to both the US and Navajo Nation agencies and provide data to the agencies [17]. The University of Pennsylvania SRP Center has developed relationships with the US EPA and interacts with them when working with the BoRit Superfund Site to facilitate information transfer between scientists and the community.

### 3.3. Technology Transfer

It is important that each center identifies opportunities and mechanisms for the transfer of biomedical and environmental science and engineering technologies generated by the projects into the hands of an end-user. This assures that the most up to date, cost-effective, and productive technology is being used in the field. Researchers at the University of California-Davis SRP Center developed an immunoassay for the detection of fipronil [18] that was brought to light by a number of companies and nonprofit organizations from Europe, China, and the U.S. when they requested reagents for evaluation or for development of other detection methods. The UCSD SRP Center licensed the reagents to a company for commercialization. A small business has also stemmed from technology developed at the UC Davis SRP, which was a patented sensor to detect mercury contamination in the environment [19].

### 3.4. Information Dissemination to Other End-Users

SRP centers not only disseminate their information within the SRP but also to multiple end-users and stakeholders not previously mentioned. The Massachusetts Institute of Technology (MIT) SRP Center publishes a blog written by their researchers and students about their research topics; however, the description of the research is written in a way that is easily understood by those that are not well versed in these scientific areas [20]. The University of Rhode Island SRP Center translates its research to target communities in their area affected by poly- and perfluoroalkyl substances in the form of easy to read handouts that they disseminate at community events but also keep on a dedicated section of their website for anyone to access [21]. The Michigan State University SRP Center translates its work to other end-users via hosting short educational courses. For example, they recently held a course that taught people about the principles of physiologically based pharmacokinetic modeling and the application of this technique in chemical health risk assessment. The research translation and community engagement teams at MSU were a major part in the planning of the 17^th^ Pediatric Research Day, which focused on environmental health effects to children and was held at a farmer’s market and translated their research for pediatricians, the public, and other stakeholders involved in the research day. To share their research with a broader community, the Texas A&M University SRP Center brought together members of the emergency response community to share valuable tips on planning ahead for possible hazards of exposures to chemicals related to natural disasters, which is the major theme of their center [22].

## 4. Conclusions

The SRP supports multidisciplinary, innovative research that advances environmental health sciences related to hazardous substance exposure and supports the protection of human health. The SRP accomplishes this through its university-based centers which provide practical and scientific solutions to develop, test, and implement unique approaches to address complex environmental health problems. Community engagement and research translation are integral in communicating, promoting, and disseminating research from the centers. The SRP ensures that the research it supports is reciprocated in an easy to understand manner to the individuals and communities that can utilize and benefit from the research. By encouraging SRP grantees to focus on developing and sharing risk prevention messages with their community partners, it allows for the basic research findings to be translated into actions that improve public health.
